# ’We discovered new ways of learning’: a qualitative study of child, caregiver, and facilitator perspectives on an inclusive school readiness programme for caregivers in Bangladesh, Nepal, and Tanzania

**DOI:** 10.1080/16549716.2026.2672307

**Published:** 2026-05-14

**Authors:** Jaya Chandna, Rejina Gurung, Mohammad Abdul Awal Miah, Jitihada Baraka, Monira Aktar, Donat Shamba, Honey Malla, Proma Paul, Rachel Lassman, Karim Manji, Raafat Hassan, Mustafa Miraji, Adori Khatun, Salma Khatun, Cally Tann, Ashish Kc, Jena Derakhshani Hamadani, Nahya Salim Masoud, Joy E. Lawn, Paul Lynch

**Affiliations:** a Maternal, Adolescent, Reproductive and Child Health (MARCH), London School of Hygiene and Tropical Medicine, London, UK; b Research Division, Golden Community, Lalitpur, Nepal; c Maternal and Child Health Division, International Centre for Diarrhoeal Disease Research, Dhaka, Bangladesh; d Department of Health Systems Impact Evaluation and Policy, Ifakara Health Institute, Dar es Salaam, Tanzania; e Department of Paediatrics and Child Health, Muhimbili University of Health and Allied Sciences, Dar es Salaam, Tanzania; f Department of Paediatrics and Child Health, Temeke Regional Referral Hospital, Dar es Salaam, Tanzania; g School of Public Health and Community Medicine, University of Gothenburg, Gothenburg, Sweden; h School of Education, University of Glasgow, Glasgow, UK

**Keywords:** School readiness, early childhood development, neuro-developmental disability, group intervention, caregiver training program

## Abstract

**Background:**

Children with disabilities face many barriers to early childhood development services and to pre-primary education. Evidence on inclusive school readiness and community-based approaches in low- and middle-income countries (LMICs) still remains limited.

**Objective:**

To explore the feasibility and acceptability of a co-designed training programme for caregivers to support school readiness among children aged 4–6 years in Bangladesh, Nepal, and Tanzania.

**Methods:**

We conducted an evaluation using 16 focus group discussions with caregivers including those with children with disabilities and facilitators who participated in the programme. Analysis drew on Ecological Systems Theory to interpret influences at child, caregiver (microsystem/mesosystem), and facilitator (exosystem) levels.

**Results:**

Participants reported gains in children’s early learning and eagerness to attend school. Caregivers described increased confidence, more responsive and positive parenting, and greater advocacy for their child’s needs. Facilitators reported improved capability to use inclusive techniques with caregivers. Three design features underpinned the perceived impact: explicit inclusive content on supporting children with additional support needs; participatory, play-based sessions co-facilitated by parent–teacher pairs; and low-cost, locally adapted materials. Reported challenges included variable attendance and punctuality, heterogeneity of children’s needs within sessions, and transport and weather disruptions.

**Conclusions:**

A co-designed, school readiness training programme for caregivers was acceptable and engaging across three LMIC settings and was perceived to strengthen child learning, caregiver practices, and facilitator skills. Embedding such programmes within communities may accelerate equitable school readiness; however, ongoing training, supervision, and attention to contextual barriers are needed for sustained impact whilst scaling-up.

## Paper Context

- **Main findings:** A co-designed, inclusive school readiness parenting group programme was feasible and acceptable in Bangladesh, Nepal and Tanzania, with perceived gains in children’s engagement and routines and caregiver practices, enabled by participatory coaching and low/no-cost adaptations despite attendance and mixed-ability challenges.
- **Added knowledge:** This study is significant in showing how participatory, play-based parenting programmes may promote school readiness for all children in LMICs, with qualitative insights that help to explain the effects of a cluster Randomized Control Trial for all children not previously attending preschool.
- **Global health impact for policy and action:** Evidence supports targeted delivery of this intervention to vulnerable children who are prevented from accessing preschool and successful embedding of inclusive school readiness caregiver programmes within government pre-primary and parenting systems to achieve equitable replication and scale-up.

## Background

Globally, over 50 million children under five experience mild-to-severe disabilities, most living in low- and middle-income countries (LMICs), with around 30 million affected by moderate-to-severe conditions [1,2]. Childhood disabilities are diverse in nature and severity, often associated with functional difficulties, such as motor, sensory impairments, communication, autism (neurodivergence), or epilepsy [3]. We define childhood neuro-developmental disability as a group of disorders in which the development of the nervous system is disturbed. This can include externalising behavioural problems (including attention deficit hyperactivity disorder (ADHD)), impaired or delayed motor and language, intellectual disabilities, and non-verbal (social) communication difficulties (autism). EN-REACH defines NDD/D based on functional difficulties using the UNICEF/Washington Group Child Functioning Module (CFM), assessing vision, hearing, mobility, self-care, communication, learning, memory, behaviour, and social interactions. Children with ‘significant difficulty’ or who are ‘unable to perform’ tasks in at least one area are classified as having moderate-to-severe functional difficulties. Many children with disabilities remain undetected during the early years of development when early identification and interventions could have the greatest impact. Barriers to early identification include community stigma, discrimination, limited training of frontline health workers in identification of NDD at this young age, lack of specialist diagnostic services, and weak referral pathways. Children with disabilities face disproportionate disadvantages, including higher risk of morbidity, premature death, lower rates of school enrolment and completion, and social exclusion [4,5]. At the same time, many children in LMICs – whether they have a diagnosed special educational need or require additional support – require different types of intervention, e.g. spectacles or low vision devices for a visual impairment, sign language, and/or hearing devices for hard of hearing or deaf [6]. Responsive parent–child relationships, supported by families and communities, are recognised as crucial for promoting successful ECD and school readiness for all children [7]. Optimal early childhood development support has lifelong benefits for educational achievement, adult productivity, and population health [8].

This paper reports on a qualitative evaluation of the acceptability and feasibility of the EN-REACH intervention, focusing on facilitator and caregiver perspectives of an innovative co-designed caregiver programme for children aged 4–6 years in Bangladesh, Nepal, and Tanzania. The new programme was designed to promote school readiness for all children, with explicit attention to the inclusion of children with developmental delays or disabilities. This qualitative evaluation was nested within the EN-REACH cluster randomised controlled trial (cRCT) [9]; full trial design and methods are described in the published protocol.

We hypothesised that a culturally adapted school readiness training programme for caregivers could improve learning outcomes among children aged 4–6 years, including those with functional difficulties (e.g. walking, communicating, behaviour), compared with a control group. School readiness is recognised as a multi-faceted construct [9], comprising several dimensions: the skills necessary to benefit from schooling, the knowledge children are expected to acquire, and guidelines on how and when these competencies should emerge [10].

The EN-REACH training package was co-designed with families, technical experts, safeguarding professionals, and education stakeholders through the use of participatory workshops. Materials were adapted from UNICEF’s Early Learning and Development Standards [11] and the Baby Ubuntu programme [12], with further input from country education experts and study teams. The intervention addressed play and stimulation, early literacy and numeracy, nutrition, communication, and preparing school bag skills. It was delivered either in community or school settings by trained parent–teacher pairs, comprising nine modules held fortnightly (2–3 h each) over a five-month period delivered to groups of between 10 and 12 caregivers and children. Extensive piloting in all three countries ensured cultural appropriateness, for example, by incorporating children’s own drawings into session materials.

Ecological Systems Theory (EST) was used to guide the evaluation, examining the perspectives of caregivers and facilitators across multiple levels: the *microsystem* (family, peers, schools), the *mesosystem* (connections between settings such as home and training sessions), and the *exosystem* (wider community and institutional influences) [13].

## Methods

### Study design and settings

We conducted a qualitative study nested within the EN-REACH cluster randomised controlled trial evaluating an inclusive school readiness parent training programme in Bangladesh, Nepal, and Tanzania. The study was carried out in accordance with the principles of the Declaration of Helsinki. The qualitative component explored programme suitability, engagement, and perceived effects among caregivers and facilitators across diverse rural and peri-urban communities.

### Intervention overview

The EN-REACH inclusive school readiness training programme for caregivers was co-designed with caregivers, teachers, and representatives from health and education. Content covered responsive caregiving, play-based early learning, positive behaviour support, and pathways for inclusion and school transition for all children using participatory and play-based approaches. Learning materials were adapted to the country context (e.g. urban, per-urban, rural) and delivered to small groups of caregivers in community locations, e.g. schools, clinics by trained facilitator pairs (a teacher and a caregiver/parent). The EN-REACH trial builds on existing cohort studies in Bangladesh, Nepal, and Tanzania, leveraging data from the already published EN-BIRTH and EN-SMILING studies, which track child development using app-based tools. The study is a subset of the EN-BIRTH/EN-SMILING cohort conducted in Bangladesh, Nepal, and Tanzania. Each cluster included 8–15 eligible children, with 50 clusters in Bangladesh, 54 in Nepal, and 56 in Tanzania. Clusters were randomly assigned (1:1) to either the EN-REACH intervention or standard of care (SOC) arm using a computer-generated randomisation list created by a statistician who was not involved in the trial.

### Participants

This study was a cRCT that recruited preschool-aged children (4–6 years) and their parents from geospatially defined clusters (urban, peri-urban, and rural). The study is a subset of the EN-BIRTH cohort conducted in Bangladesh, Nepal, and Tanzania. The methods are detailed in a previously published trial protocol [9]. The intervention groups consisted of 50 clusters in Bangladesh, 54 in Nepal, and 56 in Tanzania and participated in nine parent group sessions over 5 months, facilitated by trained personnel. Post-intervention assessments were conducted within 2 weeks of completing the final session. Each group recruited around 10–12 caregivers in each of the three countries.

The training facilitators were parents and pre-school teachers who volunteered to the role and received a 5-day training programme given by trained master trainers. Bangladesh and Nepal recruited twice as many parents and teachers (*n* = 18 and 20) than Tanzania (*n* = 11), signifying that each Tanzania pair had to run on average three cluster groups (Tables 1 and 2).

**Table 1.** Demographic characteristics of the participants of facilitator FGDs by country and by type of participant.

	Bangladesh			Nepal			Tanzania		
	Parent (n = 8)	Teacher (n = 10)	Total (n = 18)	Parent (n = 10)	Teacher (n = 10)	Total (n = 20)	Parent (n = 5)	Teacher (n = 6)	Total (n = 11)
**Age in years (Mean)**	29.0	33.7	31.6	31.5	36.4	33.9	34.0	33.0	33.5
**Gender**									
Female	8 (100.0%)	10 (100.0%)	18 (100.0%)	10 (100.0%)	10 (100.0%)	20 (100.0%)	3 (60%)	3 (50%)	6 (54.5%)
Male	0 (0.0%)	0 (0.0%)	0 (0.0%)	0 (0.0%)	0 (0.0%)	0 (0.0%)	2 (40%)	3 (50%)	5 (45.5%)
Education Level*									
Primary	–	–	–	–	–	–	3 (60%)	0 (0%)	3 (27.3%)
Lower secondary	1 (12.5%)	0 (0.0%)	1 (5.6%)	–	–	–	1 (20%)	2 (33.3%)	3 (27.3%)
Upper secondary	5 (62.5%)	1 (10.0%)	6 (33.3%)	10 (100.0%)	8 (80.0%)	18 (90.0%)	1 (20%)	0 (0%)	1 (9.1%)
Higher education	2 (25.0%)	9 (90.0%)	11 (61.1%)	0 (0.0%)	2 (20.0%)	2 (10.0%)	0 (0%)	4 (66.7%)	4 (36.4%)

*Education level aligned across the three countries: **Primary** includes Grade 1–5 in BD & NP and Standard 7 in TZ; **Lower secondary** includes Grades 6–9 in BD & NP and Forms 1–4 in TZ; **Upper secondary** includes Grades 10–12 in BD & NP and Form 5–6 in.

**Table 2.** Demographic characteristics of the participants of caregiver FGDs by country and by type of participant.

	Bangladesh			Nepal			Tanzania		
	Without Disability (*n* = 17)	With Disability (*n* = 15)	Total (*n* = 32)	Without Disability (*n* = 27)	With Disability (*n* = 5)	Total (*n* = 32)	Without Disability (*n* = 20)	With Disability (*n* = 9)	Total (*n* = 29)
**Age in years (Mean)**	30.2	36.3	33.0	29.7	30.0	29.8	33	33.3	33.1
**Gender**									
Female	16 (94.1%)	14 (93.3%)	30 (93.8%)	24 (88.9%)	4 (80.0%)	28 (87.5%)	13 (65%)	7 (77.8%)	20 (69%)
Male	1 (5.9%)	1 (6.7%)	2 (6.2%)	3 (11.1%)	1 (20.0%)	4 (12.5%)	7 (35%)	2 (22.2%)	9 (31%)
Education Level*									
Below Primary	0 (0.0%)	6 (40.0%)	6 (18.8%)	0 (0.0%)	1 (20.0%)	1 (3.1%)	3 (15%)	0 (0%)	3 (10.3%)
Primary	1 (5.9%)	1 (6.7%)	2 (6.3%)	3 (11.1%)	4 (80.0%)	7 (21.9%)	10 (50%)	5 (55.6%)	15 (51.7%)
Lower secondary	8 (47.1%)	6 (40.0%)	14 (43.8%)	10 (37.0%)	0 (0.0%)	10 (31.3%)	5 (25.0%)	3 (33.3%)	8 (27.6%)
Upper secondary	8 (47.1%)	1 (6.7%)	9 (28.1%)	9 (33.3%)	0 (0.0%)	9 (28.1%)	0 (0.0%)	0 (0.0%)	0 (0.0%)
Higher education	0 (0.0%0	1 (6.7%)	1 (3.1%)	5 (18.5%)	0 (0.0%)	5 (15.6%)	1 (5.0%)	0 (0%)	1 (3.4%)
Missing	–	–	–	–	–	–	1 (5.0%)	1 (11.1%)	2 (6.9%)

*Education level aligned across the three countries: **Below primary** includes No school or illiterate; **Primary** includes Grade 1–5 in BD & NP and Standard 7 in TZ; **Lower secondary** includes Grades 6–9 in BD & NP and Forms 1–4 in TZ; **Upper secondary** includes Grades 10–12 in BD & NP and Form 5–6 i.

### Data collection

Independent qualitative experts were identified to conduct FGDs and were then trained on the discussion guide, including the study objectives and methodology. Country teams comprised nine experienced interviewers from Bangladesh (MA, RH, AK, and SK), Nepal (PB, KTM), and Tanzania (MM, JB, SM) who used semi-structured FGD guides co-developed with stakeholders and piloted locally. FGD and interview guides explored experiences of programme delivery/participation, perceived changes for children and caregivers, inclusion of children with disabilities, and implementation barriers/enablers. FGDs were conducted in Bangla, Nepali, and Swahili by trained researchers of the same gender wherever possible, audio-recorded with consent, and accompanied by field notes. FGDs took approximately 1 h. In Nepal, audio recordings were transcribed by a research coordinator (SA), which was then proofread by the interviewer (PB and KTM) and then translated into English language by SA. In Bangladesh and Tanzania recordings were similarly transcribed and translated by experienced researchers (KTM, MA, MM). Transcripts were translated into English by an experienced bilingual translator (KTM, JB, AM) with spot-checks carried out by a second translator (MM, SM, KTM) to verify semantic and conceptual equivalence, and resolve any discrepancies by consensus.

### Research team

Multidisciplinary teams comprising experts in education, public and community child health and psychology, led the qualitative data collection and analysis in each country (seven hold a PhD and six have medical degrees). Data collection teams who visited the field in the three countries received training in inclusion, participatory methods, and how to promote participant reflexivity. Positionality statements were developed before fieldwork; debriefs after each FGD examined potential power dynamics (e.g. teacher–caregiver relationships) and how researcher backgrounds might influence questioning and interpretation.

### Data analysis

Members of the multi-disciplinary team (PL, JC, RG, JB, AM, MAAM, SM) undertook a reflexive thematic analysis, informed by Ecological Systems Theory to interpret influences at child (microsystem), caregiver/family–school interface (mesosystem), and facilitator/programme (exosystem) levels. Country teams open-coded a common subset of transcripts and co-constructed a shared codebook (Table 3). To calibrate interpretations, PL and JC independently coded 20–30% of transcripts per country in NVivo 12, compared readings and resolved differences through discussion, after which the agreed codebook was applied to remaining transcripts. We used constant comparison across countries and participant groups and maintained an audit trail (codebooks, memos, analytic decisions). Data sufficiency was assessed iteratively; no new codes emerged in the final two FGDs per country.

**Table 3.** Summary of emerging themes, categories, and sub-categories according to the level of perspective by child, caregiver, and facilitator.

Themes	Categories	Sub-categories
**Child level (microsystem)**		
Early Learning and Engagement	Improved numeracy and literacy Interested in going to school	Drawing and colouring Increased interest in writing Reading and listening to story books
Social and Behaviour change	Improved communication Positive behaviour at home and with friends	Language skills Less screen time in mobile Good behaviour with friends Making friends and greeting elders Involved in household chores Less conflicts during play
Daily living skills and safety	Improved daily habits Awareness on road safety	Active participation Handwashing, brushing, dressing, eating Healthy food choices School bag preparation
Children with disabilities		Early numeracy skills Engagement with books Emotion regulation Willingness to join group
**Caregiver and home-school practices (Mesosystem)**		
Acceptability and perceived value	Safe and non-judgemental learning space	Able to share parenting experiences Learned transferrable skills to home routines Improved understanding of other children’s feelings
Responsive positive parenting	Shift from punitive measures to play-based stimulation	Stopped punishing children Playing, making toys and reading books with children Praising children Spending time with children
Disability Inclusion Barriers	Greater confidence communicating with teachers about the special needs of children with disabilities	Letting teachers know the needs of children with disabilities at school Understanding the rights of children includes all
	Irregular attendance due to social and personal factors	Responsibility of household tasks Neighbours and families questioned involvement Time commitment for attending sessions
**Facilitator capability and implementation (Exosystem)**		
Skill, confidence and co-facilitation	Increased confidence Complementary roles within teacher–parent pairs	Used child-centred, inclusive methods Actively engaged caregivers in different role play scenarios One typically led content and modelling while the other managed children Constant communicating Ensured continuity in the absence of one facilitator
Inclusion in practice	Role shift from ‘teaching children only’ to preparing both caregivers and children	Modelling positive, non-punitive behaviour management Adapted activities for children with disabilities Emphasized the participation of children with disabilities
Professional development and interpersonal skill	Confident public speaking and management skills	Received training manual Constant mentoring by master trainers Repeated practice for engaging children with disabilities Problem solving skills Facilitating group discussion
Working with caregivers to extend learning at home	Reinforcing strategies in daily routines Adopting similar approaches they modelled for caregivers:	Counting objects while cooking, naming shapes and colours Practising calm responses to challenging behaviour.
Enablers of session quality	Shared responsibility of preparing for school, supporting parent, and engaging with children	Participatory, play-based design Inclusion of specific inclusive content for children with additional support needs Use of low/no-cost materials
Disability inclusion and attitudes	Motivated caregivers for inclusive education Greater independence with routines Sustained impact	Challenge stigma Normalise/increase participation of children with disabilities Continued home practice Supportive teacher communication
		Mixed-ability groups Continuous adjustment for pacing Late arrivals and early departures Difficult attention for two-hour sessions Transport and weather (rain/heat)

### Ethics

All signed or thumb-printed consent forms were stored in lockable filing cabinets in the lead partner country offices. Ethical approvals were obtained from institutional and national review boards – LSHTM (28166), Bangladesh: PR-221 39, Nepal: MOU 17.07.22, Tanzania: REC-02-2023-1530. Identifiers were removed during transcription; audio files and transcripts were stored on secure servers with restricted access.

## Results

Results are presented across three levels of the ecological systems framework: (1) child-level outcomes; (2) caregiver and home–school practices; (3) facilitator capability and implementation factors. Illustrative quotations are included with standardised identifiers (e.g. FGD_BD_Caregiver02_NDC = Bangladesh, caregiver, non-disabled child).

### Participant characteristics

All parents and teachers in Bangladesh and Nepal were female, and five of the parents and teachers in Tanzania were male (Table 2). The average age of the parent facilitators was 32 years and 33 for the teacher facilitators in all countries. Eleven of the Bangladeshi facilitators had received a Batchelor’s degree, whereas only four in Tanzania and two in Nepal had either a diploma or a Batchelor’s degree (Table 1).

We conducted a total of 16 focus group discussions (FGDs) in all three countries: altogether 64 caregivers of children without disabilities, 29 caregivers of children with disabilities, and 48 teacher and parent facilitators (Table 4). All sessions were conducted in schools and health centres. Recruitment was supported by school staff and community workers not involved in data collection to reduce selection bias. All participants across the three countries received a ‘school-readiness’ package comprising a set of laminated key statement cards from each session, a storybook, colouring pencils, and a small toy. The package was given to each caregiver to practise key skills they acquired during the training.

**Table 4.** Distribution of focus group discussions in the qualitative evaluation by country and by type of participant.

Participant	Number of FGD (*N* = Number of participant)			Total
	Bangladesh	Nepal	Tanzania	
Caregivers of children without disability	2 (*N* = 17)	3 (*N* = 27)	2 (*N* = 20)	93
Caregivers of children with disability	2 (*N* = 15)	1 (*N* = 5)	1 (*N* = 9)	
Teacher facilitators	1 (*N* = 9)	1 (*N* = 10)	1 (*N* = 6)	48
Parent facilitators	1 (*N* = 8)	1 (*N* = 10)	1 (*N* = 5)	

Caregivers who had a child with a disability were generally older (between 3 months and 6 years) than caregivers who had children without a disability. FGDs in Bangladesh and Nepal had significantly higher number of female caregivers than male caregivers, whereas in Tanzania, nine male caregivers attended two FGDs. In terms of education, Nepali caregivers who had children without a disability had the highest recorded level, with 19 having completed secondary school (lower and upper) and five having achieved higher degree level, e.g. Batchelor’s degree. In Nepal, all five caregivers who had a child with a disability had completed primary level only. In Bangladesh, six caregivers who have a child with a disability were recorded as not having an education at all.

## Child level (microsystem)

### Early learning and engagement

In Tanzania, caregivers described children as showing greater interest in storybooks, drawing, counting, and problem-solving games after the sessions. Several reported children initiating activities at home and persisting longer with tasks such as writing the alphabet and counting numbers.

I am really grateful that my son, who was not initially focused on writing, surprised me by showing a constant desire to write after attending the sessions. *FGD_TZ_Caregiver02_NDC*

In Nepal, caregivers reported children’s improved memory of alphabets and numbers and a shift in children’s interests from excessive use of parents’ mobiles to listening to stories told by their caregivers and playing games with siblings and friends. In Bangladesh, a small number of children were found to be able to read, sing, and write the alphabet in both English and Bangla. In addition, there was an increased interest in reading books:

After attending this session, he has become very interested in going to school. Now, he actually wants to go. When it comes to studying, he says, ‘Ammu (mom), teach me now.’ He wants to read—these interests have started to develop. *FGD_BD_Caregiver03_ CWD*

### Social and behavioural changes

Caregivers in Nepal noted more turn-taking, sharing, and respectful communication, with fewer conflicts at home and during play. Reduced shyness and greater participation in group activities were also frequently reported

After coming here, he slowly started talking to other friends, otherwise he would not talk at all. *FGD_NP_Caregiver03_NDC*

A caregiver from Bangladesh was surprised to hear her child singing along with the other children:

…… Especially the song - ‘amra korbo joy’ means ‘we will win’ that my child learned and sings this song very well, standing around with many children in my house. *FGD_BD_caregiver02_NDC*

### Daily living skills and safety

Children across the three countries increasingly managed routines (handwashing, toothbrushing, dressing, and eating) and organised school materials independently. Caregivers described initial hesitation in allowing children to perform these activities on their own, as children often made a mess and required extra cleaning, which increased caregivers’ workload.

A caregiver from Bangladesh noted how this also allowed them to have time to eat themselves:

….my child eats rice and spread while eating, so I can feed myself. After leaving the child to he is slowly eating on his own. My child can dress on his own.…… . Alhamdulillah [Thank God] now my child has learned a lot. *FGD_BD_Caregiver02_NDC*

My child can now read and write, and he keeps his books organized. He can read independently and pack his school bag after studying, which he couldn’t do before. *FGD_BD_Caregiver06_CWD*

There was also better awareness about road safety and the understanding of traffic rules when crossing streets. As one caregiver of a child with disabilities from Bangladesh said:

‘My baby has changed a lot. when he first got out on the road, he would run [away]. He never looked at the cars on the road. He was not afraid of accidents. But now, when I go out, he does not run’. *FGD_BD_Caregiver04_CWD*

### Children with disabilities

Caregivers of children with disabilities in Bangladesh reported similar gains, especially in engagement with books, acquiring early numeracy skills, and fine-motor tasks (e.g. grasping crayons, tracing letters).

Earlier, he could not hold the pen. He did not know how to hold a pen. *FGD_BD_Caregiver02_CWD*

Emotional regulation and willingness to join group play improved when activities were broken into smaller steps and positively reinforced. Facilitators also noted changes in participation of children with disabilities over the course of the sessions:

I remember a disabled child, who was very sick, has changed a lot after coming to the sessions. At first, he didn’t want to mix with anyone and used to cry. He used to bother his mother a lot, but by the next session, he would go crazy at home wanting to come. *FGD_BD_TeacherFacilitaor_CWD*.

## Caregiver and home-school practices (mesosystem)

### Acceptability and perceived value

Caregivers in Tanzania described the programme as a safe and non-judgemental space to share experiences and learn practical strategies. Despite the effort to attend, most felt sessions were worthwhile and transferrable to home routines.

‘It has made me more aware of how to better support my child’s learning and development, and I now feel more equipped to help them succeed……’ *FGD_TZN_Caregiver04_CWD*

In Bangladesh, a caregiver talked about how her daughter, who has an emotional difficulty, used to show little sympathy towards other children who got hurt while playing. Thanks to the training, she got better at understanding other children’s feelings:

… my daughter didn’t know anything before, but now she has learned many things … now she shows care and says, ‘Did you get hurt? You’ll get well soon, let’s play again. *FGD_BD_Caregiver02_CWD*

Caregivers considered the training they received as a ‘safe space’ to be able to openly share their lived experiences of raising a child with a disability without feeling judged or undermined.

One female caregiver in Bangladesh said:

… coming to the session is to learn a lot. We come here to open up the brain of our child so that we can send our children to school well. *FGD_BD_Caregiver01_CWD*

### Responsive positive parenting

Caregivers in Tanzania reported shifting from punitive responses to praise, routines, and play-based stimulation. Some described consciously spending dedicated time talking and playing with their child each day.

I realized that punishing him didn’t really help. Instead, I learned to be more patient and supportive with my child. *FGD_TZN_Caregiver05_NDC*

Now, I spend much more time with my child(ren) at home, and I make a point to set aside time to play with them and talk. *FGD_TZN_Caregiver01_CWD*

Similarly, a caregiver of a child with a disability from Nepal reflected on how she can turn some of the household objects into toys for her children:

In our house, we make toys out of small things and play with them. I have always known this, but I never passed it on to my children. I’m not sure why I didn’t pay more attention to it. Now, I do. *FGD_NP_Caregiver04_CWD*

### Inclusion of children with disability

Caregivers of children with disabilities reported greater confidence in communicating with teachers about reasonable adaptations (e.g. toileting, classroom seating, task breakdown). In Nepal, one caregiver who had previously been unable to discuss her child’s toileting needs with the class teacher later felt more confident addressing the issue directly.

Before the session, I felt embarrassed. I could not say to the teacher, ‘can you take care of my son in this way?” I used to put extra trousers in his bag … I could not request them to take him to the toilet frequently, But after the session, I always say that this is how my child is, and this is what he needs from you. *FGD_NP_Caregiver05_CWD*

Not all caregivers could regularly attend training sessions. Competing domestic work and time pressure limited participation and follow-through between sessions. Criticism from others in the household also reduced participation.

You go to the session for doing unnecessary things, leaving your household chores … *FGD_BD_Caregover06_CWD*

Several caregivers noted that routines and positive behaviours slipped when the programme ended, being described as a loss of momentum without weekly structure and reminders.

… due to the closure of the classes, he is slowly doing the same [behaviour] again. Now, even he does not want to brush in the morning … *FGD_BD_Caregiver03_CWD*

## Facilitator capability and implementation (exosystem)

### Skills, confidence, and co-facilitation

Facilitators from all three countries reported increased confidence in using child-centred, participatory approaches and actively engaging caregivers in different role-play scenarios.

They described complementary roles within teacher–parent pairs that smoothed delivery and reduced potential power imbalances: one typically led content and modelling, while the other managed children, materials, and logistics, helping sessions keep time and sustain participation. Constant communication between the facilitators between sessions also ensured continuity if one facilitator was absent for a session:

She [parent facilitator] helped me in such a beautiful way. One day I couldn’t take the session because my daughter was very ill … After visiting the doctor, I could come, and until then she ran the session. *FGD_BD_TeacherFaciltator07*

Previously we knew the traditional way of teaching … we have discovered enjoyable ways of learning through games, stories, songs and rhymes. *FGD_BD_TeacherFacilitator03*

### Professional development and interpersonal skills

The training manual was constantly monitored by master trainers (PL, JC, MAAM, RG, HM, MM, NSM), and repeated practice with groups filled knowledge gaps – especially for newer teachers who had limited experience of childhood disability.

Facilitators described growing more confident in public speaking, managing groups, prompting interaction, problem-solving, and guiding discussions about transition to school:

… we had a lack of knowledge in this regard, which this training helped to fill. *FGD_BD_TeacherFacilitator05*

With improved communication and interaction skills, we conduct sessions in a fun way that lets participants learn and share. *FGD_NP_TeacherFacilitator03*

### Working with caregivers to extend learning at home

Facilitators stressed that caregiver engagement was critical to reinforcing strategies in daily routines – counting objects while cooking, naming shapes and colours, and practising calm responses to challenging behaviour. Some facilitators described their own shift away from punishment by adopting similar approaches they modelled for caregivers:

I used to get angry and hit my daughter when she misbehaved. After training, I try to understand why she is making mistakes, and I can control my anger. *FGD_BD_ParentFacilitator08*

### Enablers of session quality

Three features were consistently cited as enabling positive outcomes at home: (i) participatory, play-based design; (ii) inclusion of specific inclusive content for children with additional support needs; and (iii) use of low/no-cost materials:

A child can be taught in many ways without a book—for example, counting vegetables or leaves while the mother is chopping. *FGD_BD_TeacherFacilitator*

Facilitators also emphasised the shared responsibility of preparing for school, supporting caregivers, and engaging with children to achieve positive school readiness

It is important to recognise the significance of a prepared school, a supportive parent, and an engaged child. *FGD_TZN_ParentFacitator03*

### Disability inclusion in practice

Facilitators emphasised modelling adapting activities so children with additional learning support needs could participate alongside peers (e.g. breaking tasks into smaller steps, offering visual supports, flexible seating to help with posture) and positive, non-punitive behaviour management. Several highlighted a shift in their role from ‘teaching children only’ to preparing both caregivers and children for home to school transition:

… previously I was just a teacher but after receiving the training I became responsible for readying both mother and child for school—reducing fear and preparing the child for school. *FGD_BD_TeacherFacilitator08*

We saw that children with disabilities can learn through play in the same group with simple adaptations. *FGD_NP_ParentFacilitator04*

Facilitators described using sessions to challenge stigma and normalise/increase participation of children with disabilities, which they felt encouraged caregivers to support schooling and inclusion:

Children should be allowed to mix with everyone … everyone should be convinced they have the right to learn and live freely. *FGD_BD_TeacherFacilitator02*

Perceived benefits were reported for children with and without disabilities. Caregivers commonly described heightened motivation to start or return to preschool and greater independence with routines. Sustained impact appeared to depend on continued home practice, supportive teacher communication, and relief of structural barriers (time, transport, seasonality).

Delivery was harder in mixed-ability groups; pacing and attention required continuous adjustment. Facilitators cautioned caregivers not to force writing when children were stressed.

There was a child at zero level, another writing A, B, C, D-two types had to be taught in two ways … We reminded parents they can’t force children to write when stressed. *FGD_NP_TeacherFacilitator02*

Some of the sessions were affected by late arrivals and early departures, and maintaining attention near the end of the 2-h sessions was difficult. Transport and weather (rain/heat) also constrained attendance.

At the beginning many mothers did not arrive on time … Waiting delayed the start and made it hard to keep attention until the end. *FGD_BD_TeacherFacilitator06*

## Discussion

This multi-country qualitative study provides new evidence that a co-designed and an inclusive school readiness programme for caregivers – delivered through participatory, play-based sessions in community or school settings – was well accepted across Bangladesh, Nepal, and Tanzania. It was valued by families, especially for those with a child with disability. Both caregivers and facilitators perceived improvements in children’s early learning, socialising, and daily routines, alongside shifts towards responsive, non-punitive caregiving and more purposeful communication between home and programme staff. Delivery of the caregiver training programme was supported by explicit disability content and simple adaptations to low or no-cost materials; common challenges included variable attendance, transport and weather disruptions, session length, and managing mixed-ability groups. Caregivers also described slippage in routines after the programme ended, indicating a need for light-touch maintenance supports wherever possible.

The qualitative findings add important depth to the EN-REACH cRCT, which found small, statistical non-significant differences in school readiness and learning outcomes between intervention and control groups. Through caregivers’ and facilitators’ narratives, this study revealed many positive perceived changes – such as increased children’s engagement, greater confidence, improved routines, and more responsive caregiving – that were not fully captured by the quantitative tools, e.g. Measuring Early Learning Quality Outcomes (MELQO). These insights suggest that the programme may have supported domains of development and inclusion not easily measured by standard school-readiness instruments, e.g. Ages & Stages.

Perceived benefits were not fully captured in the quantitative outcomes, e.g. acquisition of pre-literacy and numeracy skills. This could be due to limitations with quantitative ‘school readiness’ tools which cannot measure small changes in this age group. Caregivers of children with disabilities emphasised the value of their child being included in sessions, thus gaining confidence, and being recognised by peers and facilitators – something not easily captured by standardised quantitative child development and learning tools. This underscores the importance of an evaluation using FGDs as it can illuminate domains of change that conventional quantitative tools, such as questionnaires, may miss, especially for marginalised groups.

Our results align with evidence that caregiver-focused ECD interventions can strengthen responsive caregiving and foundational learning in LMICs [14–16] and contribute to the literature by centring disability inclusion through the use of feasible classroom/home arrangements (task breakdown, use of visual supports, flexible seating for posture for children with physical disabilities) which are consistent with inclusive pedagogy [17] and the Nurturing Care Framework [18]. Accounts of moving away from punitive responses towards positive parenting echo global guidance and research linking corporal punishment to poorer outcomes [19]. Co-facilitation appeared to facilitate partnership with caregivers and to sustain engagement, while also sharing out facilitation tasks.

Strengths of this study include comparable perspectives across the three countries considering the same tools and collaborative thematic analyses. The successful application of a theory-informed analysis using Ecological Systems Theory [20] allowed triangulation of insights from the caregiver and facilitator. Co-design and local adaptation were likely to have enabled cultural fit. Limitations include possible selection bias among FGD participants, social desirability in reporting positive findings, and variability in attendance that may shape experiences, although this was mitigated by selecting caregivers who had varied attendance, and overall attendance was extremely high (>90%). Overall, attendance rates were below 50% during the first 2–3 sessions, but a joint decision was made by each country team to give follow-up calls by country teams to remind caregivers about the forthcoming session. This intervention led to a significant increase in the number of sessions attended as well as modest help with transportation. The replication of caregiver training programmes would require careful planning on when and where the training took place and what transport means they can use to attend the sessions.

Programmatically, several implications emerge. Importantly, we have shown that inclusive education content for children with special educational needs or additional learning support needs can be integrated within mainstream caregiver curricula rather than delivered in parallel or as an add-on. Simple, low/no-cost materials and everyday activities can help to operationalise inclusion under considerable resource constraints. When mixed-ability groups are the norm, straightforward differentiation tools (e.g. activity ladders, visual schedules) can help with the delivery of a more inclusive training. Predictable barriers (seasonality, transport, caregiving workload at home) warrant flexible scheduling and brief make-up or take-home prompts, with consideration of modest transport support where feasible which can be helpful to increase attendance.

Early interest from the three government partners, who have been involved throughout in adapting EN-REACH materials, may signal a path to scale in routine platforms. For instance, the Bangladesh government has integrated the translated and culturally relevant training programme into future ECD programmes. In a publication under review, we have applied the RE-AIM framework for a detailed process evaluation to inform substantiality pathways in these three countries.

Future implementation research is needed to inform larger scale up, such as integration within government systems, testing delivery models aligned with ministry workflows (pre-primary early learning standards, supervision, monitoring), and examining how the intervention performs under routine conditions. To preserve gains after programmes end, trials could evaluate light-touch maintenance strategies such as SMS nudges or periodic booster sessions [21]. Economic evaluations are needed to inform planning and costing including for training, supervision, materials, and transport.

## Conclusions

The EN-REACH, co-designed inclusive school readiness training programme for caregivers, was acceptable across three LMIC contexts and reported to be valued by children, caregivers, and facilitators, being perceived to strengthen children’s engagement and caregiver practices and was especially valuable for those with a disability who participated. Our qualitative data give insights on how the intervention could work – through everyday, play-based routines and more responsive caregiving – and consequently help to interpret the trial pattern of statistically strongest benefit for children without prior pre-primary school attendance. In this study, we have been able to demonstrate how a short, culturally situated, school readiness training package for caregivers can create significant changes to the lives of those children who have had limited or no access to preschool. The study has also shown that prompt, well-targeted training can help families of children with disabilities to understand their specific caring and learning needs in order to prepare them for a successful transition from home into the school system.

## Supplementary Material

COREQ_Checklist_ENREACH.pdf

## Data Availability

The PI (Joy Lawn) and Co-PIs from each country will have access to the trial data. The dataset generated from the trial will be made available upon responsible request from https://datacompass.lshtm.ac.uk/

## EN-REACH collaborative group

**Bangladesh:** Mohammad Abdul Awal Miah, Shafiqul Ameen, Ismat Ara Mili, A.K.M. Tanvir Hossain, Asraful Alam, MD. Ziaul Haque Shaikh, Md. Nazmul Hasan, Mst Salma Khatun, Ms Adori Khatun, Monira Aktar, Atique Iqbal Chowdhury, Ahmed Ehsanur Rahman, Shams El Arifeen, and Jena Derakhshani Hamadani

**Nepal:** Rejina Gurung, Omkar Basnet, Chudamani Poudel, Basanta Prasad Koirala, Shova Kumari Adhikari, Arjun Dhakal, Dhanasudhan Chaulagain, Prof. Bharat Khatri, Dr Ram Chandra Bastola and Ashish KC

**Tanzania:** Nahya Salim Masoud, Mustafa Miraji, Donat Shamba, Josephine Shabani, Mohamed Bakari, Hajra Kizibo, Mohamed Akida, Aisha Mfinanga, Hellena Mariki, Ramadhani Gunda, Seif Bakari, Jitihada Baraka and Karim Manji

**UK:** Jaya Chandna, Proma Paul, Kate Mackinnon Milner, Rachel Lassman, Maria Zurmond, Cally Tann, Paul Lynch, and Joy E Lawn
